# Development and validation of a modified SOFA score for mortality prediction in candidemia patients

**DOI:** 10.1038/s41598-025-04786-8

**Published:** 2025-07-01

**Authors:** Xiaofei Liu, Ranran Ding, Guangming Yang, Yuling Qiao, Zhen Ma, Yaping Feng, Feng Qu, Qiang Meng

**Affiliations:** Department of Intensive Care Unit, Jining NO. 1 People’s Hospital, Jiankang road 6, Jining, 272000 Shandong China

**Keywords:** Candidemia, Sequential organ failure assessment, Lactate, BUN, Albumin, Experimental models of disease, Risk factors

## Abstract

Candidemia is a life-threatening bloodstream infection associated with high mortality rates, particularly in critically ill patients. Accurate risk stratification is crucial for timely intervention and could improve patient outcomes. This study aimed to enhance the predictive performance of the sequential organ failure assessment (SOFA) score by developing a modified SOFA (mSOFA) score, which is specifically designed for candidemia patients. Using data from MIMIC-III, MIMIC-IV, and ICU-JN databases, we identified key prognostic variables through LASSO regression and integrated into the mSOFA_3 model. The model incorporated respiratory_SOFA, coagulation_SOFA, and circulatory_SOFA along with clinical biomarkers, including lactate, albumin, and blood urea nitrogen. The mSOFA_3 model demonstrated superior predictive performance across multiple machine learning algorithms, with the logistic regression-based model achieving the highest AUC of 0.826 in the internal validation cohort and 0.813 in the test cohort. Kaplan-Meier survival analysis further validated the model’s utility in stratifying patients into high-risk and low-risk groups with distinct survival outcomes. These findings highlight the mSOFA_3 as a robust and clinically relevant tool for early risk stratification, offering potential for improved decision-making and therapeutic management in critically ill patients with candidemia.

**Supplementary Information:**

The online version contains supplementary material available at 10.1038/s41598-025-04786-8.

## Introduction

Candidemia is a severe and life-threatening bloodstream infection caused by *Candida* species, predominantly affecting critically ill patients in intensive care unit (ICU). Despite advancements in antifungal therapy and supportive care, the mortality rate associated with candidemia remains high, ranging between 30% and 60%^1,2^. Early diagnosis and timely initiation of appropriate antifungal therapy are critical to improving outcomes in candidemia. However, these efforts are often hindered by the nonspecific clinical presentation, delayed laboratory confirmation, and increasing antifungal resistance^3,4^. Consequently, there is a growing need for reliable prognostic tools to predict patient outcomes more accurately, guide clinical decision-making, and optimize treatment strategies.

In clinical practice, the acute physiology and chronic health evaluation II (APACHE II) score is widely recognized for its ability to predict mortality risk in critically ill patients, including those with candidemia^5^. However, its complexity and reliance on numerous variables limit its feasibility for routine use in ICU settings. In contrast, the sequential organ failure assessment (SOFA) score, designed to evaluate organ dysfunction and mortality risk in sepsis patients, offers a simpler alternative. Despite its broad utility, the predictive performance of SOFA in candidemia is suboptimal, as it may not fully capture the unique metabolic and inflammatory disturbances associated with fungal infections^6^.

To address these limitations, previous studies have attempted to enhance the SOFA score by combining it with comorbidity indices, such as the charlson comorbidity index (CCI), or by integrating additional biomarkers^7^. However, these approaches often increase complexity without sufficiently addressing candidemia-specific challenges, such as systemic inflammation, tissue hypoperfusion, and renal stress caused by fungal burden. Recent evidence highlights the potential value of infection-relevant biomarkers, such as lactate, blood urea nitrogen (BUN), and albumin, which reflect key aspects of systemic metabolic stress, tissue hypoxia, and nutritional status, respectively^8,9^. When integrated into a scoring system, these markers could enhance mortality risk prediction in candidemia patients.

In this study, we developed a modified SOFA (mSOFA) score optimized specifically for candidemia. By replacing renal_SOFA with BUN, a more sensitive marker of renal dysfunction and systemic metabolic stress, and incorporating lactate and albumin, we aimed to address the limitations of the original SOFA score. The mSOFA score integrates these biomarkers alongside key SOFA subcomponents, such as respiratory_SOFA, circulatory_SOFA, and coagulation_SOFA, as well as bicarbonate and age. Using data from the MIMIC-III and MIMIC-IV databases for model training and internal validation, and the ICU-JN database for test validation, we employed advanced statistical and machine learning techniques to construct and evaluate the model. This study enhances the SOFA score to reflect the unique pathophysiological and clinical features of candidemia in critically ill ICU patients, offering a more reliable and clinically applicable tool for guiding early risk assessment and intervention.

## Methods

### Data sources

The study data was obtained from two large open-source databases: MIMIC-III (v 1.4) and MIMIC-IV (v 1.0), and one local dataset: Jining NO.1 People’s Hospital ICU (ICU-JN) database. The MIMIC-III version 1.4 contains comprehensive records for 46,520 patients admitted to Beth Israel Deaconess Medical Center in Boston, Massachusetts, from June 2001 to October 2012 ^10^. The MIMIC-IV version 2.0 includes data on nearly 300,000 patients admitted to the same center between 2008 and 2019^11^. To address the partial overlap between MIMIC-III and MIMIC-IV datasets, we extracted data from the MIMIC-IV database for patients admitted from 2012 to 2019, while utilizing MIMIC-III data from 2001 to 2008 to ensure comprehensive coverage^10^. The relevant clinical data from these databases include patient demographics, vital signs, laboratory results, microbial culture results, medication, procedure records, and survival information. The users must complete a collaborative training course provided by the US National Institutes of Health. After passing the “Protecting Human Research Participants” training course on the National Institutes of Health (NIH) website, the author (Qiang Meng) received approval to extract data from the database for research purposes (certification number: 56251014). Both MIMIC-III and MIMIC-IV are publicly available and anonymized, thus exempting them from additional ethical committee approval. The ICU-JN database is sourced from the Dong Hua electronic medical record system of Jining No. 1 People’s Hospital, covering the period from 1th December 2020, to 31th January 2024. Data collection from the ICU-JN dataset was approved by the institutional review board of Jining No. 1 People’s Hospital (approval reference number KYLL-202308-134). The ethics review board at Ji Ning No.1 people’s Hospital granted an exemption from the acquisition of informed consent, given the retrospective nature of the study. All methods were performed in accordance with the relevant guidelines and regulations, including the Declaration of Helsinki.

### Study population

The study focused on critically ill adult patients diagnosed with candidemia, defined as the presence of *Candida* species in at least one blood culture, accompanied by clinical signs and symptoms of infection. The inclusion criteria were: (1) age ≥ 18 years when entering the ICU; (2) patients with complete SOFA score data. The exclusion criteria were: (1) length of stay in the ICU < 24 h; (2) patients who co-infected with bacteremia within 7 days before and after the detection of candidemia; (3) patients who were pregnant or lactating. For those who had multiple ICU admissions with candidemia, only the first admission was included.

### Data extraction and study design

Navicat software (v 15.1) was used for data extraction, while STATA software (v 18.0) was employed to concatenate records based on the hadm_id, stay_id or icustay_id codes. The detailed process of data extraction is illustrated in Fig. 2. We extracted the following variables measured within the first 24 h after the diagnosis of candidemia: (1) demographic information at admission: age and gender; (2) vital signs within 24 h: heart rate (HR)_max, heart rate (HR)_min, systolic blood pressure (SBP)_min, diastolic blood pressure (DBP)_min, mean arterial pressure (MAP)_min, temperature_max, and respiratory rate (RR)_max, respiratory rate (RR)_min and SPO_2__min; (3) laboratory results: white blood cell count (WBC)_max, hemoglobin_min, platelet count_min, lactate_max, PaO_2_/FiO_2__min, bicarbonate_min, partial prothrombin time(PTT)_max, international normalized ratio (INR)_max, prothrombin time(PT)_max, blood urea nitrogen(BUN)_max, creatinine_max, alanine aminotransferase (ALT)_max, aspartate aminotransferase(AST)_max, bilirubin_max; (4) vasoactive drug administration within 24 h: dopamine, epinephrine, norepinephrine, dobutamine, and vasopressin; (5) antifungal treatments initiated: voriconazole, amphotericin B, caspofungin, posaconazole; (6) Comorbidities, including hypertension, chronic obstructive pulmonary disease, diabetes, myocardial infarction, and solid tumors, were identified using the international classification of diseases, ninth revision (ICD-9), along with the tenth revision (ICD-10) diagnosis codes when discharge; and (7) components of the maximum sequential organ failure assessment (SOFA) score recorded within 24 h: respiratory_score, coagulation_score, liver_score, cardiovascular_score, glasgow coma scale (GCS)_score, and renal_score. The primary outcome of this study is the 28-day mortality following the time of blood culture collection after candidemia diagnosis. If no death was documented within 28 days of the candidemia diagnosis, the patient was classified as a survivor.

**Fig. 1:**
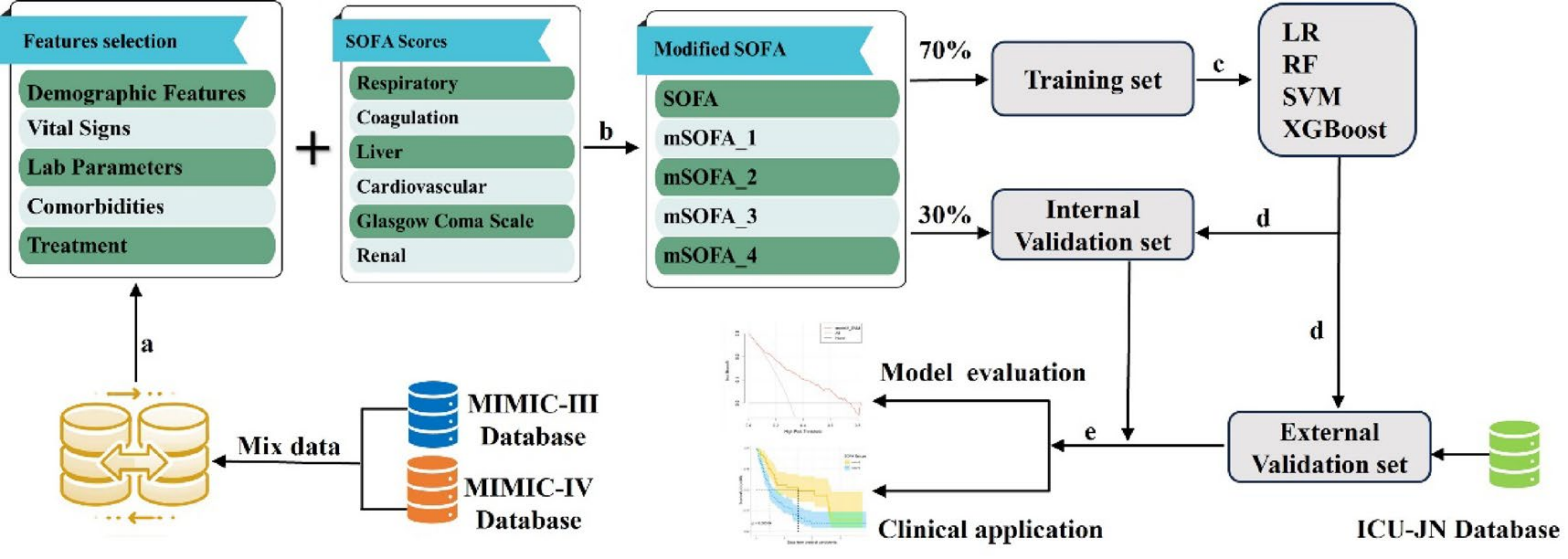
Overview of the Study Design. (**a**) Univariate and multivariate analyses of clinical data for variable selection; (**b**) LASSO analysis using the selected clinical variables combined with the SOFA score for further variable selection to construct a modified SOFA score models; (**c**) Model training using Logistic Regression (LR), Random Forest (RF), Support Vector Machine (SVM), and Extreme Gradient Boosting (XGBoost) algorithms; (**d**) Model validation of internal and test cohorts; (**e**) Model evaluation and clinical application.

The clinical data were obtained by combining datasets from the MIMIC-III and MIMIC-IV databases (Fig. 1). Univariate analysis was first conducted using logistic regression to identify significant prognostic variables (*p* < 0.05). The selected variables were subsequently included in multivariate logistic regression analysis to determine their independent prognostic value (Fig. 1a). The significant predictors, along with relevant components of the SOFA score, were utilized to construct a modified SOFA score aimed at enhancing predictive accuracy for patient outcomes in candidemia. Following this, the clinical data underwent standardization, while the individual components of the SOFA score remained unchanged, and least absolute shrinkage and selection operator (LASSO) analysis was conducted to identify key variables for the modified SOFA (mSOFA) score (Fig. 1b). The dataset for the mSOFA score was randomly divided into a training cohort, which included 70% of the selected admissions, and an internal validation cohort, comprising the remaining 30%. The data from the ICU-JN dataset was employed as an external test set to evaluate the model’s generalizability. Following this division, clinical variables were assessed and scored based on their clinical relevance. Variables identified through LASSO analysis, including both the clinically scored variables and original SOFA score components, were then used to construct four mSOFA models with different combinations, aiming to determine the most accurate model for predicting mortality risk in candidemia patients. After constructing multiple mSOFA models, we evaluated each model’s performance using random forest (RF), logistic regression (LR), support vector machine (SVM), and extreme gradient boosting (XGBoost) algorithms (Fig. 1c). For each model, we generated receiver operating characteristic (ROC) curves and calculated the area under the curve (AUC) to identify the model with the highest AUC as the optimal predictive model (Fig. 1d). After model training, we performed a calibration curve analysis to assess the agreement between predicted and observed mortality risks. Additionally, decision curve analysis (DCA) was also conducted to evaluate the clinical utility of the model across a range of threshold probabilities. Finally, survival analysis was performed to further explore the prognostic value of the model for candidemia patients (Fig. 1e).

In our study, we employed Templ’s visualization method (R Package “VIM”)^12^ to recognize the distribution of missing values. For cases with over 50% missing data and incomplete SOFA scores were removed. Besides, variables with more than 30% missing values were removed, such as D-dimer, procalcitonin, pH_min, pH_max, glucose_max, glucose_min and C-reaction protein. The missing value percentage of all variables were provided in Supplementary Table S1. Multiple imputation was applied to missing values in the remaining data using the ‘mice’ package of R software^13^.

### Statistical analysis

Descriptive statistics were computed for all categorical and continuous variables. To assess the comparability of the training, validation, and test cohorts, we compared the overall distribution of candidate variables across the three datasets. Categorical variables were presented as frequencies and percentages, while continuous variables were summarized as means with standard deviations or medians with interquartile ranges, depending on their distribution. The chi-squared test or fisher’s exact test was used to compare categorical variables, and Student’s t-test or Mann-Whitney U test was applied for continuous variables, as appropriate. For three-group comparisons, one-way analysis of variance (ANOVA) was used for normally distributed variables, and the Kruskal–Wallis test was used for non-normally distributed variables.

Univariate and multivariate logistic regression analyses were performed to identify significant predictors of mortality in candidemia patients. The results of these analyses were used to select relevant clinical features for inclusion in the modified SOFA score model. All statistical analyses were performed using R software (v 4.1.2). *p*<0.05 was considered statistically significant.

### Model development and selection

Following data preprocessing, a five-fold cross-validated LASSO analysis was applied to identify the most predictive features from the selected clinical variables and the original SOFA score components. LASSO analysis introduces an L1 penalty, which shrinks coefficients of less significant variables to zero, aiding in feature selection and preventing overfitting. Using the ‘glmnet’ package in R, the optimal lambda parameter was determined by minimizing the cross-validated error, thus achieving a balance between model fit and complexity.

Next, we combined the selected variables in different ways and constructed multiple predictive models through random forest (RF), logistic regression (LR), support vector machine (SVM), and extreme gradient boosting (XGBoost) algorithms to determine the optimal model. Each model’s hyperparameters were fine-tuned via ten folds cross-validation, ensuring robust selection of parameters (details are provided in Supplementary Appendix). These models were trained on the training cohort and evaluated on the internal validation and test cohorts. Model performance was assessed using multiple evaluation metrics, including the area under the receiver operating characteristic curve (AUC), F1 score, recall, accuracy, and precision. The model with the highest AUC was selected as the optimal model.

### Model evaluation and application

Calibration curves and decision curve analysis (DCA) were plotted to assess the model’s accuracy and clinical utility, respectively. The selected model was validated using calibration curves to compare predicted and observed mortality outcomes. Decision curve analysis (DCA) was conducted to evaluate the net clinical benefit of the model across a range of threshold probabilities^14,15^. Finally, survival analysis was performed using the Kaplan-Meier method, categorizing patients into high-risk and low-risk groups based on the mSOFA score. The survival differences between these groups were evaluated using the log-rank test, with a statistically significant result (*p* < 0.05). All statistical analyses were performed using R software (v 4.1.2).

## Results

### Baseline characteristics

**Fig. 2:**
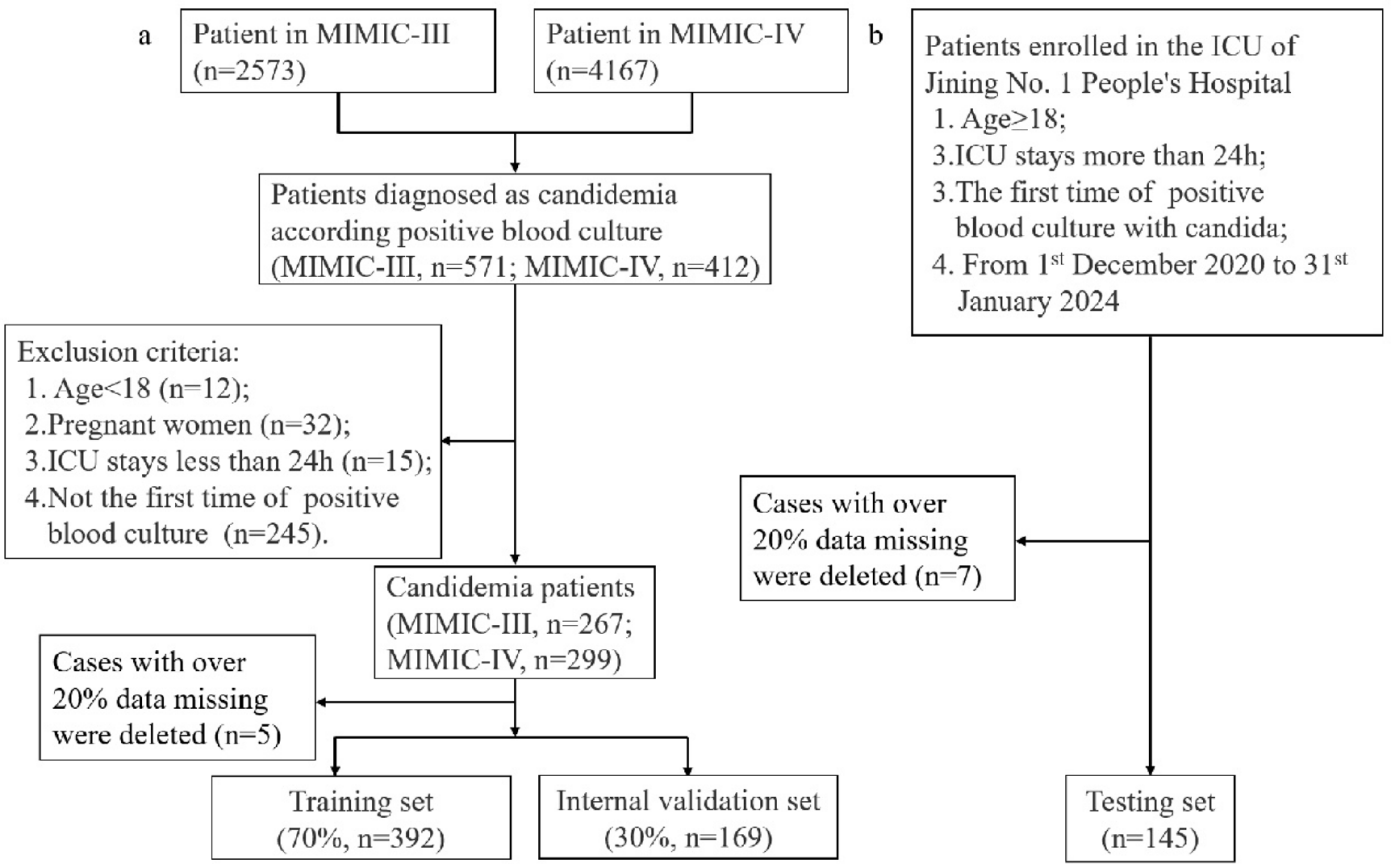
Flowchart of patient selection. (**a**) Patients select from MIMIC-III and MIMIC-IV databases for model training and internal validation sets. (**b**) Patients select from Jining NO. 1 people’s hospital for testing set. MIMIC-III: medical information mart for intensive care-III; MIMIC-IV: medical information mart for intensive care-IV; ICU: intensive care unit.

This study included a total of 561 candidemia patients, with 267 cases obtained from the MIMIC-III database and 299 cases from the MIMIC-IV database. For test validation, data of 145 patients with candidemia were collected from the ICU-JN database, based at Jining No. 1 People’s Hospital. The patients from the MIMIC-III and MIMIC-IV databases were randomly divided into a training cohort (*n* = 392) and an internal validation cohort (*n* = 169) at a 7:3 ratio. The detailed procedure for patient selection is provided in Fig. 2.

A comparison of baseline characteristics and univariable analysis between survival and death candidemia patients in the MIMIC -III and MIMIC-IV databases is summarized in Table 1. The 28-day all-cause mortality rate for patients with candidemia was 62.21% (349/561). Non-survivors had a significantly higher mean age (64.16 ± 15.55 years) compared to survivors (58.09 ± 15.64 years). Non-survivors had significantly lower temperature_max (37.78 and 38.15℃), lower MBP (45.98 and 51.43 mmHg) and higher rates of vasopressor use (75.6% vs. 55.7%), indicating hemodynamic instability, while heart rate, respiratory rate, oxygen saturation, and PaO₂/FiO₂ ratios showed no significant differences between groups. Significant abnormalities in blood coagulation indexes of non-survivors, such as prolonged PTT (63.20 and 40.55 s) and PT time (17.30 and 14.85 s), higher INR (1.60 and 1.30), and lower platelet count (137.12 × 10^9^/L and 198.96 × 10^9^/L). Comorbidities appeared to have a limited impact on mortality risk in candidemia. Non-survivors exhibited more pronounced organ dysfunction, as evidenced by significantly higher levels of ALT (758.37 and 304.65 IU/L), AST (2041.05 and 611.09 IU/L), lactate (3.70 and 2.20 mmol/L), and BUN (61.64 and 36.72 mg/dl), and lower serum albumin (2.31 and 2.66 mg/dL), compared to survivors (*p* < 0.05 for each).

**Table 1:** Baseline characteristics and univariable analysis for 28-day all-cause mortality candidemia patients.

Variables	Survival (*n* = 212)	Death (*n* = 349)	OR	CI	*P* value
**Demographic**					
age (years, mean (SD))	58.09 (15.64)	64.16 (15.55)	1.02	1.01–1.04	< 0.001
gender (male, n (%))	127 (59.9)	188 (53.9)	1.28	0.9–1.81	0.162
**Vital signs** ^a^					
temperature_max (℃, mean(SD))	38.15 (0.90)	37.78 (1.01)	0.67	0.56–0.8	< 0.001
RR_max (times/min, mean (SD))	33.39 (8.06)	31.64 (7.33)	0.97	0.95–0.99	0.009
RR_min (times/min, mean (SD))	12.00 (4.98)	12.20 (4.90)	1.01	0.97–1.04	0.633
SBP_min (mmHg, mean (SD))	83.09 (16.61)	72.69 (22.24)	0.97	0.96–0.98	< 0.001
DBP_min (mmHg, mean (SD))	40.89 (12.36)	35.58 (12.05)	0.96	0.95–0.98	< 0.001
MBP_min (mmHg, mean (SD))	51.43 (16.27)	45.98 (16.62)	0.98	0.97–0.99	< 0.001
HR_max (times/min, mean (SD))	122.15 (24.12)	119.19 (24.95)	1	0.99 -1	0.169
HR_min (times/min, mean (SD))	73.67 (17.17)	73.56 (19.50)	1	0.99–1.01	0.946
SPO_2__min (%, median (IQR))	90.00 (81.00, 93.00)	90.50 (87.00, 93.00)	0.98	0.96–0.99	0.052
**Laboratory tests** ^b^					
Bilirubin_total_max (mg/dl, mean (SD))	2.12 (4.73)	3.56 (6.82)	1.05	1.01–1.1	0.016
Albumin_min (mg/dL, mean (SD))	2.66 (0.67)	2.31 (0.65)	0.45	0.34–0.59	< 0.001
PaO_2_/FiO_2__min (mmHg, mean (SD))	237.53 (105.68)	214.71 (110.71)	1.01	1-1.02	0.030
ALT_max (IU/L, mean (SD))	304.65 (22.47)	758.37 (59.86)	1.02	1.01–1.03	0.006
AST_max (IU/L, mean (SD))	611.09 (150.23)	2041.05 (527.39)	0.98	0.94–1.02	0.008
BUN_max (mg/dl, mean (SD))	36.72 (28.20)	61.64 (34.18)	1.03	1.02–1.04	< 0.001
Bicarbonate_max (mg/dl, mean (SD))	26.17 (4.80)	25.17 (6.19)	0.97	0.94 -1	0.046
Bicarbonate_min (mg/dl, mean (SD))	22.21 (4.43)	17.83 (5.37)	0.83	0.8–0.87	< 0.001
Creatinine (mg/dl, median (IQR))	1.00 (0.70, 1.70)	1.30 (0.70, 2.40)	1.12	0.98–1.29	0.086
Lactate_max (mmol/L, median (IQR))	2.20 (1.60, 3.42)	3.70 (2.10, 7.60)	1.29	1.2–1.4	< 0.001
WBC_max (×10^9^, mean (SD))	14.93 (11.17)	16.01 (19.72)	1.77	1.21–2.6	0.522
WBC_min (×10^9^, mean (SD))	6.50 (3.63)	5.43 (3.45)	1.01	0.97–1.04	0.732
Hemoglobin_max (g/dl, mean (SD))	9.16 (1.84)	8.74 (2.07)	0.9	0.83–0.98	0.018
Hemoglobin (g/dl, mean (SD))	8.83 (1.45)	9.00 (1.78)	1.24	1.12–1.37	0.298
Platelet_max (×10^9^, mean (SD))	372.27 (186.48)	284.03 (190.41)	1.01	1 -1.02	< 0.001
Platelet_min (×10^9^, mean (SD))	198.96 (111.27)	137.12 (107.42)	0.99	0.99 -1.0	< 0.001
Calcium_max (mean (SD))	8.61 (0.83)	8.95 (1.11)	1.42	1.18–1.7	< 0.001
Calcium_min (mean (SD))	7.87 (0.81)	7.65 (0.98)	0.77	0.63–0.93	0.006
Potassium_max (mean (SD))	4.52 (0.80)	4.94 (0.85)	1.94	1.53–2.46	< 0.001
Potassium_min (mean (SD))	3.70 (0.58)	3.61 (0.66)	0.8	0.61–1.04	0.097
Fibrinogen (mg/dl, mean (SD))	348.34 (175.30)	350.69 (194.82)	1.54	1.2–1.98	0.897
INR_max (median (IQR))	1.30 (1.20, 1.70)	1.60 (1.40, 2.20)	1.03	1-1.06	< 0.001
PT_max (sec, median (IQR))	14.85 (13.30, 18.22)	17.30 (14.70, 22.90)	1.01	0.99–1.03	< 0.001
PTT_max (sec, median (IQR))	40.55 (32.00, 86.05)	63.20 (40.50, 115.40)	0.17	0.11–0.26	< 0.001
**Comorbidities**					
COPD (yes, n (%))	12 (5.7)	15 (4.3)	0.75	0.34–1.63	0.466
Diabetes (yes, n (%))	68 (32.1)	111 (31.8)	0.99	0.68–1.42	0.947
Coronary (yes, n (%))	30 (14.2)	41 (11.7)	0.81	0.49–1.34	0.407
Hypertension (yes, n (%))	62 (29.2)	100 (28.7)	0.97	0.67–1.42	0.881
Tumor (yes, n (%))	4 (1.9)	6 (1.7)	0.91	0.25–3.26	0.884
CKD (yes, n (%))	39 (18.4)	79 (22.6)	1.32	0.78–2.23	0.295
HF (yes, n (%))	56 (26.4)	123 (35.2)	1.49	0.94–2.36	0.087
Liver disease (yes, n (%))	20 (9.4)	70 (20)	2.5	1.32–4.75	0.004
**Treatment measures**					
CVC (yes, n (%))	179 (	326 (93.4)	2.61	1.49–4.59	< 0.001
Antifungul (yes, n (%))	50 (23.6)	103 (29.5)	1.36	0.92–2.01	0.153
Vasoactive drug (yes, n (%))	118 (55.7)	264 (75.6)	1.78	0.65–4.89	0.004
**SOFA score**					
Respiratory_SOFA (mean (SD))	1.19 (1.37)	2.00 (1.46)	1.47	1.29–1.69	< 0.001
Coagulation_SOFA (mean (SD))	0.78 (1.09)	1.32 (1.29)	1.46	1.24–1.73	< 0.001
Liver_SOFA (mean (SD))	0.55 (1.07)	1.08 (1.33)	1.46	1.22–1.75	< 0.001
Cardiovascular_SOFA (mean (SD))	1.73 (1.35)	2.40 (1.47)	1.39	1.21–1.59	< 0.001
GCS_SOFA (mean (SD))	0.58 (0.97)	0.81 (1.24)	1.21	1.01–1.44	0.036
Renal_SOFA (mean (SD))	1.22 (1.44)	1.85 (1.51)	1.34	1.17–1.53	< 0.001

Categorical data were presented as frequency (percentage), parametric continuous data were presented as mean ± (standard deviation), whereas non-parametric continuous data were presented as median (interquartile ranges). CKD: chronic kidney disease; HF: heart failure; COPD: chronic obstructive pulmonary disease; SOFA: sequential organ failure assessment; GCS: glasgow coma scale; HR: heart rate; MAP: mean arterial pressure; SBP: systolic blood pressure; RR: respiratory rate; WBC: white blood cell count; PT: prothrombin time; PTT: partial thromboplastin time; INR: international normalized ratio; BUN: blood urea nitrogen; ALT: alanine aminotransferase; AST: aminotransferase. Vital signs were recorded as the maximum or minimum values within the first 24 h after blood culture collection for each included patient with candidemia. The laboratory tests recorded the worst value during the first 24 h after blood culture collection for each included patient with candidemia.

### Prediction model Building and evaluation

**Fig. 3:**
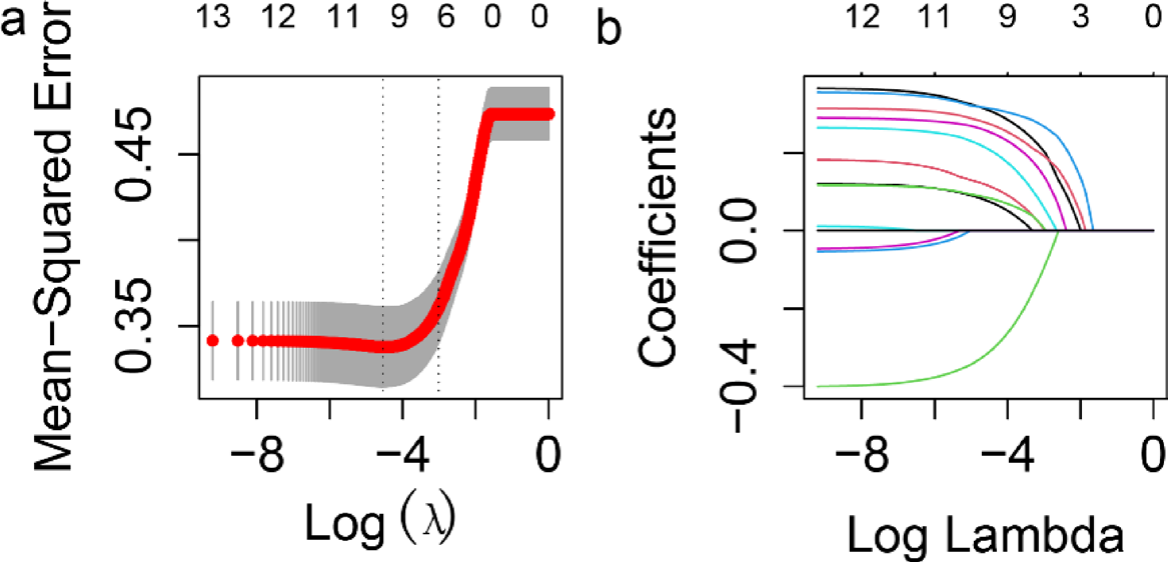
Identification of variables for the modified SOFA model using LASSO regression analysis. (**a**) LASSO regression cross-validation curve. The optimal λ value was selected using cross-validation in training cohort. (**b**) Path diagram of the LASSO coefficients. Each curve illustrates the trajectory of the coefficients for each variable as λ changes. The vertical axis represents the coefficient values, the lower horizontal axis shows log(λ), and the upper horizontal axis indicates the number of non-zero variables included in the model at each λ value.

**Fig. 4:**
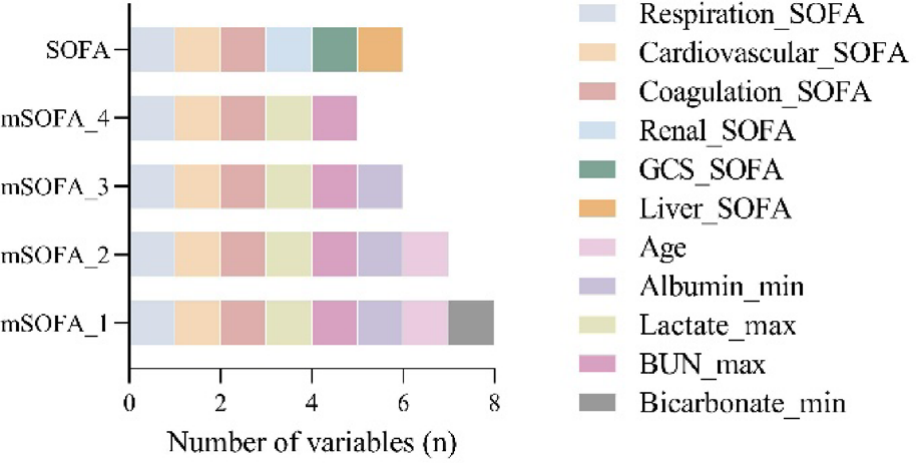
Comparison of variable combinations in different mSOFA models and the original SOFA score. The horizontal stacked bar chart illustrates the distribution of variables across mSOFA models and the SOFA score. The x-axis indicates the number of variables, while the y-axis denotes the models.

To identify predictors of mortality in candidemia patients, clinically significant variables from univariate analysis were incorporated into a multivariable logistic regression. This process identified 7 significant predictors associated with increased mortality risk: age, RR_max, temperature_max, lactate_max, bicarbonate_min, albumin_min, and BUN_max. The results of the multivariable logistic regression analysis are presented in Supplementary Table S2. The characteristics of the candidemia patients in the training cohort, internal validation cohort and test cohort were presented in Table 2. In addition to the comparisons between survival and death groups, we assessed the overall distribution of baseline characteristics across the training, internal validation, and test cohorts. The distributions of key clinical variables such as age, vital signs, laboratory indicators, and organ dysfunction scores were generally similar among the three cohorts. For example, the mean age across the three cohorts ranged from 61.6 to 62.7 years, and the levels of lactate, albumin, BUN, and SOFA scores demonstrated comparable trends. These results suggest a relatively balanced distribution of clinical features across cohorts, supporting the comparability and generalizability of our modeling (Supplementary Table S3). These variables, along with the six components of the original SOFA score, were further refined through LASSO regression to construct a modified SOFA (mSOFA) model optimized for predicting mortality in candidemia patients. Based on the optimal lambda value (lambda.min = 0.03710371), LASSO identified three original SOFA variables (respiration_SOFA, coagulation_SOFA, and cardiovascular_SOFA) and five clinical variables (BUN_max, bicarbonate_min, albumin_min, lactate_max, and age) as the most predictive factors for candidemia mortality, shown in Fig. 3. Following this selection, we constructed four distinct mSOFA models using different combinations of these variables. Details of these models are provided in Fig. 4.

**Table 2:** Variables of mSOFA in the training, validation and test sets.

Variables	Training set (*n* = 392)				Validation set (*n* = 169)			Test set (*n* = 145)		
	Survival (*n* = 146)	Death (*n* = 246)		*P* value	Survival (*n* = 62)	Death (*n* = 107)	*P* value	Survival (*n* = 58)	Death (*n* = 87)	*P* value
Age (years, mean (SD))	58.52 (15.28)	64.00 (15.85)		0.001	58.05 (15.03)	64.28 (15.63)	0.022	59.22 (15.82)	69.81 (38.02)	0.021
Temperature_max (℃, mean(SD))	38.18 (0.88)	37.72 (0.99)		< 0.001	38.30 (0.86)	37.78 (1.02)	0.003	38.19 (0.96)	37.72 (0.96)	< 0.001
RR_max (times/min, mean (SD))	34.83 (8.73)	32.02 (7.84)		0.001	36.33 (8.64)	31.67 (8.17)	0.002	33.53 (9.09)	31.44 (7.61)	0.153
Lactate_max (mmol/L, median (IQR))	2.97 (2.03)	5.52 (5.04)		< 0.001	2.86 (1.80)	5.54 (4.83)	< 0.001	2.45 (1.74)	5.21 (4.62)	< 0.001
Bicarbonate_min (mg/dl, mean (SD))	22.16 (4.47)	17.64 (5.53)		< 0.001	21.78 (4.67)	17.73 (6.04)	< 0.001	21.62 (4)	18.07 (4.91)	< 0.001
Albumin_min (mg/dL, mean (SD))	2.63 (0.62)	2.27 (0.63)		< 0.001	2.68 (0.70)	2.29 (0.63)	0.001	2.67 (0.58)	2.36 (0.6)	< 0.001
BUN_max (mg/dl, mean (SD))	34.60 (25.66)	62.74 (33.62)		< 0.001	38.24 (29.56)	62.14 (32.73)	< 0.001	38.33 (31.47)	65.61 (36.14)	< 0.001
Respiratory_SOFA (mean (SD))	1.48 (1.51)	1.83 (1.56)		0.03	1.47 (1.54)	1.81 (1.56)	0.032	1.52 (1.59)	1.8 (1.63)	0.293
Coagulation_SOFA (mean (SD))	1.08 (1.17)	1.47 (1.36)		0.004	1.21 (1.25)	1.56 (1.37)	0.043	1.19 (1.26)	1.45 (1.38)	0.251
Liver_SOFA (mean (SD))	0.97 (1.30)	1.13 (1.39)		0.235	0.82 (1.05)	1.13 (1.49)	0.205	0.78 (1.17)	1.05 (1.36)	0.201
Cardiovascular_SOFA (mean (SD))	2.14 (1.44)	2.67 (1.45)		< 0.001	2.18 (1.47)	2.76 (1.40)	0.02	2.07 (1.4)	2.6 (1.52)	0.033
Renal_SOFA (mean (SD))	1.49 (1.51)	2.11 (1.55)		< 0.001	1.60 (1.51)	2.15 (1.55)	0.041	1.4 (1.51)	1.95 (1.47)	0.029
GCS_SOFA (mean (SD))	5.35 (1.51)	5.31 (1.55)		0.056	5.60 (1.51)	5.15 (1.55)	0.041	5.4 (1.51)	5.95 (1.47)	0.029

Categorical data were presented as frequency (percentage), parametric continuous data were presented as mean ± (standard deviation), whereas non-parametric continuous data were presented as median (interquartile ranges). RR: respiratory rate; BUN: blood urea nitrogen; GCS: glasgow coma scale.

**Fig. 5:**
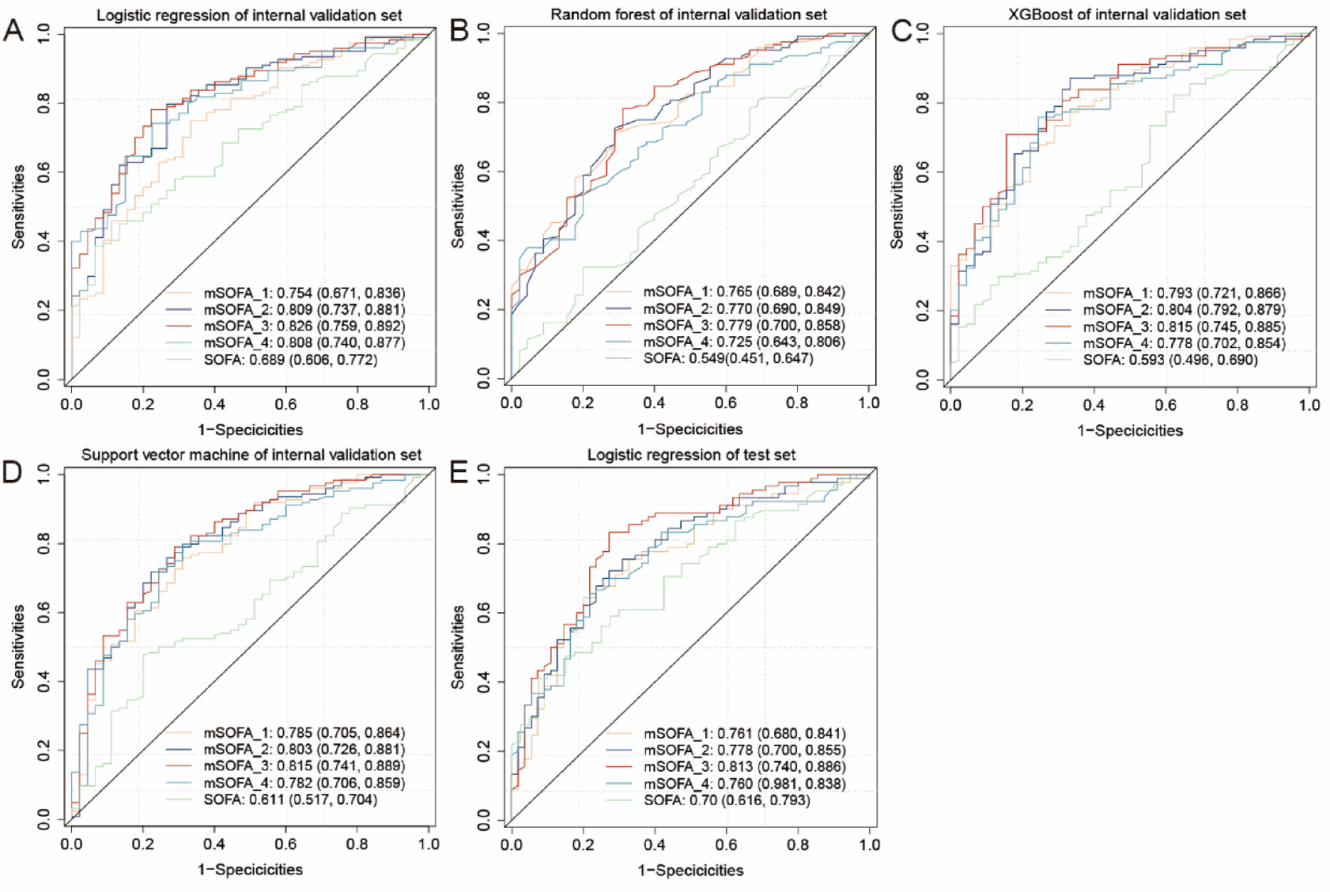
Receiver operating characteristic (ROC) curves and performance metrics for the mSOFA models. (a-d) ROC curves of the mSOFA_3 model using four different machine learning algorithms in the internal cohort: (**a**) Logistic regression (LR), (**b**) Random forest, (**c**) Extreme gradient boosting, and (**d**) Support vector machine. (**e**) ROC curve and performance metrics of the LR-based mSOFA_3 model in the test cohort (ICU-JN database).

To assess model performance, we employed four machine learning algorithms: logistic regression (LR), random forest (RF), support vector machine (SVM), and extreme gradient boosting (XGBoost). Among the four models, the LR-based mSOFA_3 model achieved the highest AUC of 0.826 (95%CI: 0.759, 0.892), F1 scores of 0.825, recall of 0.758, accuracy of 0.763, and precision of 0.904, indicating optimal predictive performance (Fig. 5a-d). The mSOFA_3 model comprised respiratory, coagulation, and circulatory components of the original SOFA score along with clinical variables lactate_max, albumin_min, and BUN_max (Fig. 4). External validation of mSOFA_3 using ICU-JN database resulted in an AUC of 0.813 (95%CI: 0.740, 0.886), F1 scores of 0.788, recall of 0.744, accuracy of 0.752, and precision of 0.838, confirming robust generalizability of the model (Fig. 5e). Detailed metrics performance of other models is presented in Supplementary Table S4.

The calibration curves for the mSOFA_3 model’s logistic regression analysis showed good agreement between predicted and observed mortality in both the internal validation cohort (Mean absolute error = 0.017) and the test cohort (Mean absolute error = 0.03), indicating reliable model calibration (Supplementary Fig. S1). To assess the clinical utility of the mSOFA_3 model, the Decision Curve Analysis (DCA) curve was plotted for both the internal validation and test cohorts (Supplementary Fig. S2). The DCA demonstrated a net benefit for clinical intervention, with threshold probability ranges of 0.15–0.85 in the internal cohort (Supplementary Fig. S2a) and 0.2–0.9 in the test cohort (Supplementary Fig. S2b). These results support the clinical utility and applicability of the mSOFA_3 model for mortality risk prediction.

### Model application

To enhance the clinical applicability of the mSOFA_3 model in predicting mortality in candidemia, we transformed lactate, BUN, and albumin values into scores ranging from 0 to 4 based on clinical significance, reflecting severity^16–18^. The original SOFA components (respiratory_SOFA, coagulation_SOFA, and cardiovascular_SOFA) remained unchanged, as detailed in Supplementary Table S5.

**Fig. 6:**
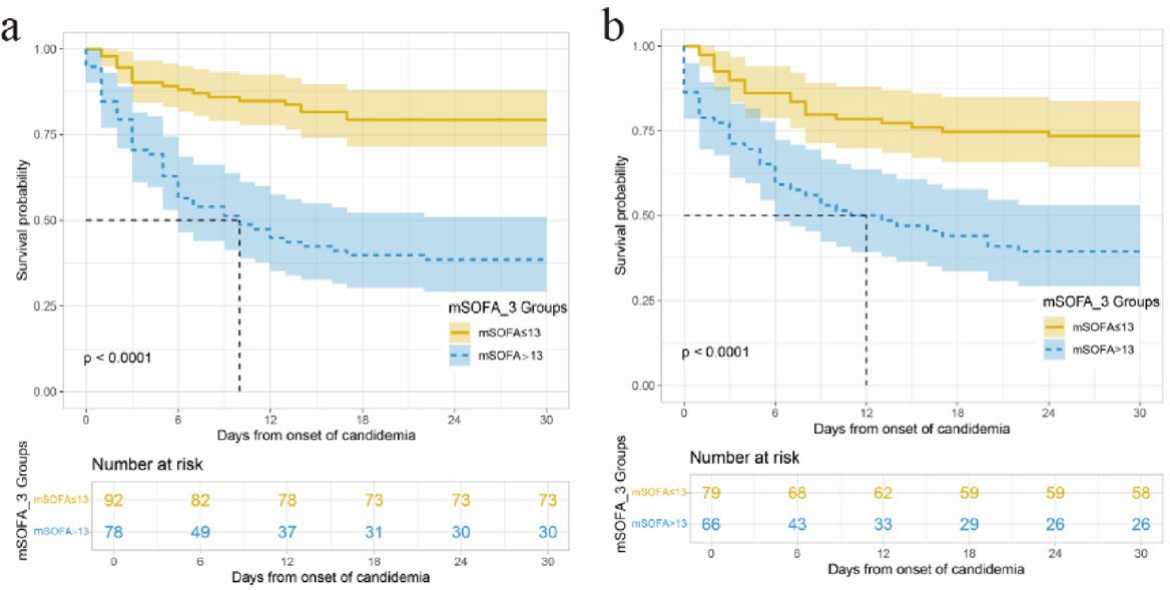
Kaplan-Meier survival curves stratified by mSOFA_3 score in internal validation and test cohorts. Patients were classified into low-risk (mSOFA ≤ 13) and high-risk (mSOFA > 13) groups. (**a**) the survival curves for the internal validation cohort, (**b**) the survival curves for the test cohort.

To further evaluate the clinical utility of the mSOFA_3 model, we performed a 28-day survival analysis. Using the median mSOFA_3 score of 13 as the cutoff, patients with a score ≤ 13 were classified as the low-risk group, and those with a score > 13 as the high-risk group. Kaplan-Meier survival curves demonstrated that the 28-day survival rate was significantly higher in the low-risk group compared to the high-risk group (Fig. 6).

## Discussion

In this study, we developed the modified SOFA (mSOFA) score as a prognostic tool to predict 28-day mortality in patients with candidemia. Considering the complexity of candidemia, a precise and reliable prognostic tool is essential for informed clinical decision-making and effective resource allocation. LASSO analysis identified eight variables for the construction of the mSOFA score, including respiratory_SOFA, circulatory_SOFA, and coagulation_SOFA components, along with five additional clinical parameters: age, lactate_max, BUN_max, albumin_min, and bicarbonate_min. Interestingly, while BUN did not exhibit a high OR in the multivariate logistic regression analysis, it was retained in the mSOFA model following LASSO selection.In contrast, liver_SOFA, renal_SOFA, and GCS_SOFA components were excluded, reflecting their relatively limited prognostic value in the context of candidemia.

This aligns with recent findings highlighting the value of BUN as a prognostic indicator in critically ill patients, particularly in the context of sepsis and candidemia^1,19^. While serum creatinine and urine output remain fundamental for diagnosing acute kidney injury (AKI) according to standard guidelines, their utility as mortality predictors in critically ill patients can be influenced by clinical interventions. Urine output may be affected by diuretics, fluid resuscitation, or renal replacement therapy^1^which may limit its reliability for mortality prediction. Although serum creatinine serves as an excellent marker of renal function, BUN provides a more comprehensive evaluation of patient status by reflecting inflammatory status, protein catabolism, and nutritional status, making it a more sensitive predictor of mortality risk in critically ill patients^20–23^. Multicenter studies have demonstrated that elevated BUN levels independently predict adverse outcomes in ICU populations, and outperform both serum creatinine and eGFR in mortality prediction^19,24^.The exclusion of liver_SOFA is consistent with previous studies that questioned the relevance of these components in specific critical care scenarios, particularly in sepsis patients^16^. Furthermore, the exclusion of GCS_SOFA underscores the potential limitations of neurological assessment in capturing mortality risk specific to candidemia, as altered mental status may not be as prominent or predictive in this patient population^25^. These adjustments collectively emphasize the importance of refining the SOFA score to prioritize variables with stronger prognostic relevance for candidemia and similar conditions.

To validate the mSOFA score, we constructed four different models and evaluated their performance using four machine learning algorithms: logistic regression (LR), random forest (RF), support vector machine (SVM), and XGBoost. Among these, the logistic regression-based mSOFA_3 score, incorporating BUN_max, lactate_max, albumin_min, and the SOFA components of respiratory_SOFA, circulatory_SOFA, and coagulation_SOFA, achieved AUC values of 0.826 and 0.815 in the internal and test cohorts, respectively. These results highlight the robust predictive capability of the mSOFA_3 model in stratifying mortality risk among candidemia patients.

Compared to the original SOFA score, the mSOFA_3 model offers several advantages. First, the removal of liver_SOFA and renal_SOFA components enhances its predictive accuracy in candidemia, as these variables are less predictive of mortality in this specific context. Second, the inclusion of non-SOFA parameters such as lactate, BUN, and albumin reflects their broader prognostic utility in critical care. Notably, the simplified scoring approach for lactate, BUN, and albumin, which mirrors the 0–4 structure of SOFA components, further improves the model’s usability in clinical practice. The exclusion of GCS_SOFA also reduces the reliance on subjective neurological assessments, making the mSOFA_3 score more objective and reproducible across different clinical settings.

Among the variables included in the mSOFA_3 score, lactate, BUN, and albumin are particularly notable. Albumin, as the most abundant serum protein in humans, serves not only as a marker of systemic inflammation and nutritional status but also as a potential neutralizer of microbial toxins. For instance, albumin has been shown to mitigate damage caused by *Candida albicans* by neutralizing its cytolytic toxin, candidalysin^26^. Low albumin levels reflect poor nutritional reserves and heightened inflammatory states, both of which are closely associated with worse clinical outcomes in critically ill patients^27^.Lactate, a well-established marker of tissue hypoxia and metabolic dysfunction, plays a critical role in identifying patients at risk of poor outcomes, especially in sepsis and other critical illnesses^28^. Elevated lactate levels indicate inadequate tissue oxygenation and cellular metabolic stress, both of which strongly correlate with poor prognosis. In candidemia, where metabolic derangements and tissue hypoperfusion are common, lactate provides crucial insights into the severity of the underlying physiological stress^8^. Similarly, BUN has emerged as a sensitive indicator of renal dysfunction and systemic metabolic stress. Unlike creatinine, BUN levels often rise earlier and more prominently in septic shock and multi-organ dysfunction, making it a more sensitive marker of mortality risk in critically ill patients^19,29,30^. In candidemia, systemic inflammation and renal hypoperfusion are common, further highlighting the value of BUN in capturing the severity of renal and overall organ dysfunction. Studies have consistently shown that elevated BUN levels are strongly associated with adverse outcomes across various critical illnesses, including sepsis, heart failure, and acute kidney injury^9,23^. Together, albumin, lactate, and BUN provide an integrated view of both metabolic and inflammatory disturbances in candidemia patients. Their inclusion in the mSOFA_3 model addresses the limitations of the original SOFA score and enhances the predictive capacity of this tailored tool for risk stratification and decision-making in this high-risk population.

To facilitate practical application, we transformed lactate, BUN, and albumin into a 0–4 scoring range based on their clinical significance, similar to the original SOFA components. This approach simplifies the calculation process, making the mSOFA_3 score more accessible for routine use. In survival analysis, patients were stratified into low-risk (mSOFA score ≤ 13) and high-risk (mSOFA score > 13) groups based on the median score. Kaplan-Meier analysis revealed significant differences in 28-day survival between these groups, with low-risk patients showing markedly better survival outcomes. These findings suggest that the mSOFA_3 model not only identifies high-risk patients but also supports clinical decision-making by providing actionable prognostic insights.

The mSOFA_3 model demonstrated key strengths in its simplicity, reproducibility, and strong performance across internal and test cohorts. However, certain limitations warrant consideration. First, while the model’s predictive accuracy was robust, further validation in larger and more diverse cohorts is needed to confirm its generalizability. Second, while our study focused on clinical variables routinely measured in candidemia patients, incorporating emerging biomarkers could potentially enhance the model’s predictive capacity. Lastly, the use of a fixed threshold (mSOFA score ≤ 13) for risk stratification may not fully capture the dynamic nature of critical illness; future studies should explore adaptive thresholds or machine learning–based approaches for further refinement.

## Conclusion

In conclusion, the mSOFA_3 model provides an effective, practical, and clinically relevant tool for predicting mortality in patients with candidemia. By integrating critical metabolic and inflammatory markers, the model surpasses the traditional SOFA score in prognostic accuracy while maintaining simplicity and ease of use. Further prospective validation and integration with decision-support systems will be crucial to fully establish its utility in clinical practice.

## Electronic supplementary material

Below is the link to the electronic supplementary material.

Supplementary Material 1

## Data Availability

The dataset utilized in this paper was acquired from the MIMIC-IV (version 1.4) database (https://physionet.org/content/mimiciii/1.4/) and MIMIC-IV (version 2.2) database (https://physionet.org/content/mimiciv/2.2/) with granted permission. The datasets used and/or analyzed during the current study can be obtained from the corresponding author (Q.M.) upon reasonable request. The code for extracting variables is available on GitHub (https://github.com/mengqiang1985/candidemia-mortality-code/tree/main).

## References

[1] Poissy, J. et al. Risk factors for candidemia: a prospective matched case-control study. *Crit. Care*. **24**, 109. 10.1186/s13054-020-2766-1 (2020). 32188500 10.1186/s13054-020-2766-1 PMC7081522

[2] Lausch, K. R. et al. High incidence of candidaemia in a nationwide cohort: underlying diseases, risk factors and mortality. *Int. J. Infect. Dis.* **76**, 58–63. 10.1016/j.ijid.2018.08.010 (2018). 30176293 10.1016/j.ijid.2018.08.010

[3] Giacobbe, D. R. et al. Early diagnosis of candidemia with explainable machine learning on automatically extracted laboratory and Microbiological data: results of the AUTO-CAND project. *Ann. Med.* **55**, 2285454. 10.1080/07853890.2023.2285454 (2023). 38010342 10.1080/07853890.2023.2285454 PMC10836245

[4] McCarty, T. P., White, C. M. & Pappas, P. G. Candidemia and invasive candidiasis. *Infect. Dis. Clin. North. Am.* **35**, 389–413. 10.1016/j.idc.2021.03.007 (2021). 34016283 10.1016/j.idc.2021.03.007

[5] Liu, F. et al. Clinical characteristics and prognostic risk factors of candidemia in non-neutropenic patients: a retrospective cohort study. *Eur. J. Clin. Microbiol. Infect. Diseases: Official Publication Eur. Soc. Clin. Microbiol.* **42**, 1389–1394. 10.1007/s10096-023-04672-z (2023). 10.1007/s10096-023-04672-z 37792119

[6] Hu, W. H., Lin, S. Y., Hu, Y. J., Huang, H. Y. & Lu, P. L. Application of machine learning for mortality prediction in patients with candidemia: feasibility verification and comparison with clinical severity scores. *Mycoses* **67**, e13667. 10.1111/myc.13667 (2024). 37914666 10.1111/myc.13667

[7] Asai, N. et al. Combination of sequential organ failure assessment (SOFA) score and Charlson comorbidity index (CCI) could predict the severity and prognosis of candidemia more accurately than the acute physiology, age, chronic health evaluation II (APACHE II) score. *BMC Infect. Dis.* **21**, 77. 10.1186/s12879-020-05719-8 (2021). 33451284 10.1186/s12879-020-05719-8 PMC7811217

[8] Kazancioglu, S. et al. Candidemia in critically ill COVID-19 patients: risk factors and impact on mortality. *Heliyon* **10**, e28033. 10.1016/j.heliyon.2024.e28033 (2024). 38545189 10.1016/j.heliyon.2024.e28033 PMC10966599

[9] Zhang, J. et al. Establishment and validation of a nomogram clinical prediction model for nosocomial candidemia: an 18-Year retrospective analysis. *Infect. Drug Resist.* **17**, 4455–4466. 10.2147/IDR.S480028 (2024). 39431215 10.2147/IDR.S480028 PMC11491067

[10] Johnson, A. E. et al. MIMIC-III, a freely accessible critical care database. *Sci. Data*. **3**, 160035. 10.1038/sdata.2016.35 (2016). 27219127 10.1038/sdata.2016.35 PMC4878278

[11] Johnson, A. E. W. et al. MIMIC-IV, a freely accessible electronic health record dataset. *Sci. Data*. **10**, 1. 10.1038/s41597-022-01899-x (2023). 36596836 10.1038/s41597-022-01899-x PMC9810617

[12] Templ, M., Alfons, A. & Filzmoser, P. Exploring incomplete data using visualization techniques. *Adv. Data Anal. Classif.* **6**, 29–47. 10.1007/s11634-011-0102-y (2012).

[13] Zhang, Z. Multiple imputation with multivariate imputation by chained equation (MICE) package. *Ann. Transl Med.* **4**, 30. 10.3978/j.issn.2305-5839.2015.12.63 (2016). 26889483 10.3978/j.issn.2305-5839.2015.12.63 PMC4731595

[14] Xie, P., Wang, W. & Dong, M. A predictive model for 30-Day mortality of fungemia in ICUs. *Infect. Drug Resist.* **15**, 7841–7852. 10.2147/IDR.S389161 (2022). 36605852 10.2147/IDR.S389161 PMC9809363

[15] Vickers, A. J., Cronin, A. M., Elkin, E. B. & Gonen, M. Extensions to decision curve analysis, a novel method for evaluating diagnostic tests, prediction models and molecular markers. *BMC Med. Inf. Decis. Mak.* **8**, 53. 10.1186/1472-6947-8-53 (2008). 10.1186/1472-6947-8-53 PMC2611975 19036144

[16] Martin-Rodriguez, F. et al. Time for a prehospital-modified sequential organ failure assessment score: an ambulance-Based cohort study. *Am. J. Emerg. Med.* **49**, 331–337. 10.1016/j.ajem.2021.06.042 (2021). 34224955 10.1016/j.ajem.2021.06.042

[17] Miano, N. et al. Controlling nutritional status (CONUT) score as a potential prognostic Indicator of In-Hospital mortality, Sepsis and length of stay in an internal medicine department. *Nutrients* **15**10.3390/nu15071554 (2023). 10.3390/nu15071554 PMC10096657 37049392

[18] Weng, J. et al. Development and validation of a score to predict mortality in ICU patients with sepsis: a multicenter retrospective study. *J. Transl Med.* **19**, 322. 10.1186/s12967-021-03005-y (2021). 34325720 10.1186/s12967-021-03005-y PMC8319895

[19] Wernly, B. et al. Blood Urea nitrogen (BUN) independently predicts mortality in critically ill patients admitted to ICU: A multicenter study. *Clin. Hemorheol Microcirc*. **69**, 123–131. 10.3233/CH-189111 (2018). 29758935 10.3233/CH-189111

[20] Li, X. et al. Association between blood Urea nitrogen and 30-day mortality in patients with sepsis: a retrospective analysis. *Ann. Palliat. Med.* **10**, 11653–11663. 10.21037/apm-21-2937 (2021). 34872290 10.21037/apm-21-2937

[21] Bozzetti, F. & Stanga, Z. Does nutrition for cancer patients feed the tumour? A clinical perspective. *Crit. Rev. Oncol. Hematol.* **153**, 103061. 10.1016/j.critrevonc.2020.103061 (2020). 32777729 10.1016/j.critrevonc.2020.103061

[22] Huq, S. et al. The prognostic impact of nutritional status on postoperative outcomes in glioblastoma. *World Neurosurg.* **146**, e865–e875. 10.1016/j.wneu.2020.11.033 (2021). 33197633 10.1016/j.wneu.2020.11.033

[23] Toda, M. et al. Population-Based active surveillance for Culture-Confirmed Candidemia - Four sites, united states, 2012–2016. *MMWR Surveill Summ.* **68**, 1–15. 10.15585/mmwr.ss6808a1 (2019). 31557145 10.15585/mmwr.ss6808a1 PMC6772189

[24] Khoury, J. et al. Blood Urea nitrogen variation upon admission and at discharge in patients with heart failure. *ESC Heart Fail.* **6**, 809–816. 10.1002/ehf2.12471 (2019). 31199082 10.1002/ehf2.12471 PMC6676277

[25] Donoso-Calero, M. I. et al. Clinical outcome prediction of acute neurological patients admitted to the emergency department: sequential organ failure assessment score and modified SOFA score. *Front. Public. Health*. **11**, 1264159. 10.3389/fpubh.2023.1264159 (2023). 37965516 10.3389/fpubh.2023.1264159 PMC10642972

[26] Austermeier, S. et al. Albumin neutralizes hydrophobic toxins and modulates Candida albicans pathogenicity. *mBio* **12**, e0053121. 10.1128/mBio.00531-21 (2021). 34154403 10.1128/mBio.00531-21 PMC8262992

[27] Li, Y. et al. Machine-learning based prediction of prognostic risk factors in patients with invasive candidiasis infection and bacterial bloodstream infection: a singled centered retrospective study. *BMC Infect. Dis.* **22**, 150. 10.1186/s12879-022-07125-8 (2022). 35152879 10.1186/s12879-022-07125-8 PMC8841094

[28] de Souza, D. C., Jabornisky, R. & Kissoon, N. Utility of lactate levels in the diagnosis and prognosis of septic shock. *Pediatr. Emerg. Care*. **40**, 736–745. 10.1097/PEC.0000000000003181 (2024). 39514790 10.1097/PEC.0000000000003181

[29] Li, J. L. et al. Assessment of clinical sepsis-associated biomarkers in a septic mouse model. *J. Int. Med. Res.* **46**, 2410–2422. 10.1177/0300060518764717 (2018). 29644918 10.1177/0300060518764717 PMC6023044

[30] Njim, T., Dondorp, A., Mukaka, M. & Ohuma, E. O. Identifying risk factors for the development of sepsis during adult severe malaria. *Malar. J.* **17**, 278. 10.1186/s12936-018-2430-2 (2018). 30064433 10.1186/s12936-018-2430-2 PMC6066934

