# Supplementary material for: Development and validation of a modified SOFA score for mortality prediction in candidemia patients
Source: Sci Rep. 2025 Jul 1;15:22044. doi: 10.1038/s41598-025-04786-8 (PMC12215393; doi:10.1038/s41598-025-04786-8)
Supplement: Supplementary file 1 — Supplementary Material 1 [file 41598_2025_4786_MOESM1_ESM.docx]

**Development and validation of a modified SOFA score for mortality prediction in candidemia patients**

Xiaofei Liu ^1^, Ranran Ding^1^, Guangming Yang^1^, Yuling Qiao^1^, Zhen Ma^1^, Yaping Feng^1^, Feng Qu^1^*, and Qiang Meng ^1^*

^1^ Department of Intensive Care Unit, Jining No. 1 People's Hospital affiliated to Shandong first medical university, Jining 272111, Shandong, China.

First authors:

Xiaofei Liu^1^ and Ranran Ding^1^. These authors contributed equally to this work and share first authorship.

*Corresponding author:

Feng Qu^1*^ and Qiang Meng^1*^. These authors made equal contributions to this work and serve as co-corresponding authors.

E-mail: rmyyzzeq@163.com (F. Q.);

E-mail: mengqiang198500@163.com (Q. M.).

**Supplementary method**

**Random forest (LR) method**

Random Forest is an ensemble learning method that combines multiple decision trees to improve predictive accuracy and control overfitting. Each tree is trained on a bootstrap sample of the data, and feature selection is randomized at each split, ensuring diversity among the trees and robust predictions.

The performance of the RF model depends on two key hyperparameters:

Number of features considered for splitting at each node (mtry): Tested values: 2, 4, 6.

Number of trees (ntree): Tested values: 100, 200, 300.

We utilized the caret package for hyperparameter tuning. A 5-fold cross-validation strategy was employed to assess model performance across different combinations of these parameters. The optimal configuration was identified based on the cross-validated accuracy.

Additionally, manual adjustment was performed for the number of trees (ntree) by fixing the best value of mtry. Each configuration was evaluated, and the results were summarized using resampling statistics. The final RF model was trained using the optimal hyperparameters (mtry = 3, ntree = 100).

**Logistic regression (LR) method**

Logistic regression is a statistical method used for binary outcomes, where the dependent variable is categorical with two possible outcomes, such as survival or mortality. We defined the logistic regression model with the following equation:

hospital_expire_flag=β0+β1(repiratory_SOFA)+β2(coagulation_SOFA)+β3(cardiovascular_SOFA)+β4(albumin)+β5(lactate)+β6(BUN)

Where:

hospital_expire_flag is the binary outcome variable (1 = patient expired, 0 = patient survived).

repiratory_SOFA, coagulation_SOFA, cardiovascular_SOFA, albumin, lactate, and BUN are the independent variables representing various clinical scores and measures.

The logistic regression model was fitted using the glm() function with a binomial family to model the binary nature of the outcome.

**Support vector machine (SVM) method**

SVM is a robust classification algorithm that constructs a hyperplane to maximize the margin between classes in high-dimensional space. The radial basis function (RBF) kernel enables the algorithm to capture non-linear relationships between input features and outcomes, making it suitable for complex datasets.

To optimize the SVM model, we conducted a grid search for two key hyperparameters:

Gamma (sigma): Controls the influence of a single training sample. Tested values: 0.1, 1, 10.

Cost (C): Determines the trade-off between achieving a low error on the training data and minimizing the model's complexity. Tested values: 0.1, 1, 10.

A 10-fold cross-validation procedure was implemented to evaluate the performance of different parameter combinations. The optimal parameters were identified based on the cross-validated performance, and the final SVM model was trained using the best hyperparameter configuration.

**Extreme gradient boosting (XGBoost) method**

XGBoost is an advanced and efficient implementation of gradient-boosted decision trees that excels in predictive accuracy and scalability. It employs a tree ensemble approach to optimize model predictions by combining multiple weak learners, where each tree contributes incrementally to reducing the overall prediction error.

The target variable was binary and predictor variables were standardized using Z-score normalization.

The initial model was trained using the logistic loss function (binary:logistic) to predict binary outcomes, with parameters such as maximum tree depth (max_depth), learning rate (eta), and feature sampling ratio (colsample_bytree) configured to enhance performance. A grid search coupled with 10-fold cross-validation was conducted to optimize key hyperparameters. The following parameters were tuned during model development:

Number of trees (nrounds): 100, 200, 300

Maximum tree depth (max_depth): 3, 5, 8

Learning rate (eta): 0.01

Minimum loss reduction for split (gamma): 0, 0.1

Subsample ratio (subsample): 0.8

Feature sampling ratio (colsample_bytree): 0.8

Minimum sum of instance weights in a leaf (min_child_weight): 1, 3

**Supplementary Tables**

| Supplementary Table S1. Summary of Missing Data in the Training and Validation Cohorts (n=561). | | | | | | | |
| --- | --- | --- | --- | --- | --- | --- | --- |
| Variables | Total counts (n) | Missing counts (n) | Missing proportion (%) | Variables | Total counts (n) | Missing counts (n) | Missing proportion (%) |
| Age | 561 | 0 | 0 | WBC_max | 561 | 88 | 15.7 |
| Gender | 561 | 0 | 0 | WBC_min | 561 | 88 | 15.7 |
| Heartrate_max | 561 | 42 | 7.4 | Platelet_max | 561 | 88 | 15.7 |
| Heartrate_min | 561 | 42 | 7.4 | Platelet_min | 561 | 88 | 15.7 |
| SBP_max | 561 | 47 | 8.3 | Hemoglobin_min | 561 | 88 | 15.7 |
| SBP_min | 561 | 47 | 8.3 | Hemoglobin_max | 561 | 88 | 15.7 |
| DBP_min | 561 | 47 | 8.3 | Calcium_max | 561 | 31 | 5.5 |
| DBP_max | 561 | 47 | 8.3 | Calcium_min | 561 | 31 | 5.5 |
| MBP_max | 561 | 47 | 8.3 | Potassium_max | 561 | 20 | 3.5 |
| MBP_min | 561 | 47 | 8.3 | Potassium_min | 561 | 20 | 3.5 |
| RR_max | 561 | 41 | 7.2 | INR_max | 561 | 31 | 5.5 |
| RR_min | 561 | 41 | 7.2 | INR_min | 561 | 31 | 5.5 |
| Temperature_max | 561 | 56 | 10.1 | PT_max | 561 | 31 | 5.5 |
| Spo2_min | 561 | 41 | 7.2 | PT_min | 561 | 31 | 5.5 |
| pH_min | 561 | 146 | 26.0 | PTT_max | 561 | 37 | 6.5 |
| pH_max | 561 | 146 | 26.0 | PTT_min | 561 | 37 | 6.5 |
| PO_2_/FiO_2__min | 561 | 94 | 16.8 | CVC | 561 | 0 | 0.0 |
| Lactate_max | 561 | 89 | 15.9 | Vasoactive drug | 561 | 0 | 0 |
| Bicarbonate_max | 561 | 21 | 3.7 | Antifungual | 561 | 0 | 0 |
| Bicarbonate_min | 561 | 21 | 3.7 | COPD | 561 | 0 | 0 |
| Glucose_max | 561 | 168 | 30.0 | Coronary | 561 | 0 | 0 |
| Glucose_min | 561 | 168 | 30.0 | Hypertension | 561 | 0 | 0 |
| ALT_max | 561 | 27 | 4.8 | Diabetes | 561 | 0 | 0 |
| AST_max | 561 | 28 | 4.9 | Tumor | 561 | 0 | 0 |
| Bilirubin_total_max | 561 | 47 | 8.3 | CRP_max | 561 | 200 | 35.6 |
| Albumin_min | 561 | 54 | 9.5 | D-dimer_max | 561 | 253 | 45.1 |
| Creatinine_max | 561 | 20 | 3.5 | BUN_max | 561 | 20 | 3.5 |

| Supplementary Table S2. Multivariable logistic regression analysis for factors potentially associated with 28-day mortality in patients with candidemia (n= 561, 160 survive, 331 death). | | | |
| --- | --- | --- | --- |
| Variables | OR | CI | *P* value |
| Age | 1.02 | 1.01-1.04 | 0.002 |
| SBP_min | 0.81 | 0.63-1.05 | 0.108 |
| DBP_min | 0.98 | 0.95-1.01 | 0.198 |
| MBP_min | 1.01 | 0.98-1.04 | 0.37 |
| RR_max | 0.96 | 0.93-0.99 | 0.01 |
| TEMP_max | 0.97 | 0.96-0.98 | <0.001 |
| SPO_2__min | 0.98 | 0.94-1.01 | 0.174 |
| PO2/FiO2 | 1 | 0.98-1.02 | 0.758 |
| Lactate_max | 1.22 | 1.11-1.34 | <0.001 |
| Bicarbonate_min | 0.86 | 0.81-0.92 | <0.001 |
| ALT_max | 1 | 0.97-1.03 | 0.614 |
| AST_max | 1 | 0.98-1.02 | 0.954 |
| Bilirubin_total_max | 1 | 0.97-1.02 | 0.756 |
| Albumin_min | 0.6 | 0.43-0.85 | 0.004 |
| BUN_max | 1.01 | 1-1.02 | 0.02 |
| Platelet_max | 1.18 | 0.97-1.54 | 0.061 |
| Platelet_min | 1 | 0.98-1.02 | 0.813 |
| Calcium_max | 1.3 | 0.99-1.71 | 0.056 |
| Calcium_min | 0.82 | 0.56-1.2 | 0.308 |
| Potassium_max | 1.06 | 0.77-1.44 | 0.729 |
| INR_max | 1.33 | 0.96-1.85 | 0.089 |
| PT_max | 0.97 | 0.93-1.01 | 0.161 |
| PTT_max | 1 | 1-1.01 | 0.754 |
| CVC | 1.12 | 0.55-2.28 | 0.746 |
| Vasoactive drug | 1.27 | 0.76-2.13 | 0.366 |

| Supplementary Table S3. Comparison of overall distributions of mSOFA-related variables among the training, validation, and test cohorts. | | | | |
| --- | --- | --- | --- | --- |
| Variables | Training set （n=392） | Validation set (n=169) | Test set (n=145) | *p* value |
| Age (years, mean (SD)) | 62.0 (15.8) | 61.6 (15.9) | 62.7 (14.9) | 0.844 |
| Temperature_max (℃, mean (SD)) | 37.9 (0.98) | 38.0 (0.99) | 37.9 (0.98) | 0.494 |
| RR_max (times/min, mean (SD)) | 33.1 (8.29) | 31.7 (8.03) | 32.3 (8.26) | 0.182 |
| Lactate_max (mmol/L, median (IQR)) | 2.9 (1.9, 5.5) | 2.8 (1.9, 5.3) | 3.1 (1.6, 4.5) | 0.533 |
| Bicarbonate_min (mg/dl, mean (SD)) | 19.3 (5.60) | 19.8 (5.11) | 19.5 (4.88) | 0.609 |
| Albumin_min (mg/dL, mean (SD)) | 2.40 (0.65) | 2.54 (0.73) | 2.48 (0.61) | 0.081 |
| BUN_max (mg/dl, mean (SD)) | 52.3 (33.7) | 52.1 (35.6) | 54.7 (36.8) | 0.742 |
| Respiratory_SOFA (mean (SD)) | 1.70 (1.55) | 1.70 (1.62) | 1.69 (1.62) | 0.997 |
| Coagulation_SOFA (mean (SD)) | 1.32 (1.30) | 1.36 (1.33) | 1.34 (1.34) | 0.951 |
| Liver_SOFA (mean (SD)) | 1.07 (1.36) | 0.95 (1.32) | 0.94 (1.29) | 0.445 |
| Cardiovascular_SOFA (mean (SD)) | 2.48 (1.47) | 2.47 (1.49) | 2.39 (1.49) | 0.809 |
| Renal_SOFA (mean (SD)) | 1.88 (1.56) | 1.82 (1.58) | 1.73 (1.51) | 0.617 |
| GCS_SOFA (mean (SD)) | 1.28 (1.46) | 1.12 (1.37) | 1.28 (1.50) | 0.444 |

| Supplementary Table S4. The prediction performance of each model in internal validation and test validation cohorts. | | | | | | | | |
| --- | --- | --- | --- | --- | --- | --- | --- | --- |
|  | Models | AUC | CI | F1 | Recall | Accuracy | Precision |  |
| LR (Internal validation cohort) | SOFA | 0.689 | 0.606, 0.772 | 0.689 | 0.589 | 0.609 | 0.829 |  |
|  | mSOFA_1 | 0.754 | 0.671, 0.836 | 0.802 | 0.750 | 0.728 | 0.861 |  |
|  | mSOFA_2 | 0.809 | 0.737, 0.881 | 0.823 | 0.766 | 0.757 | 0.888 |  |
|  | mSOFA_3 | 0.826 | 0.759，0.892 | 0.825 | 0.758 | 0.763 | 0.904 |  |
|  | mSOFA_4 | 0.808 | 0.740, 0.877 | 0.791 | 0.718 | 0.722 | 0.881 |  |
| RF (Internal validation cohort) | SOFA | 0.549 | 0.451, 0.647 | 0.323 | 0.356 | 0.604 | 0.296 |  |
|  | mSOFA_1 | 0.765 | 0.689, 0.842 | 0.5 | 0.533 | 0.716 | 0.471 |  |
|  | mSOFA_2 | 0.770 | 0.690, 0.849 | 0.505 | 0.511 | 0.734 | 0.500 |  |
|  | mSOFA_3 | 0.779 | 0.700，0.858 | 0.587 | 0.600 | 0.775 | 0.574 |  |
|  | mSOFA_4 | 0.725 | 0.643, 0.806 | 0.452 | 0.467 | 0.698 | 0.438 |  |
| SVM (Internal validation cohort) | Sofa | 0.611 | 0.517, 0.704 | 0.783 | 0.798 | 0.669 | 0.761 |  |
|  | Model1 | 0.785 | 0.705, 0.864 | 0.865 | 0.823 | 0.746 | 0.29 |  |
|  | Model2 | 0.803 | 0.726, 0.881 | 0.885 | 0.831 | 0.763 | 0.844 |  |
|  | Model3 | 0.815 | 0.741，0.889 | 0.911 | 0.71 | 0.850 | 0.793 |  |
|  | Model4 | 0.782 | 0.706, 0.859 | 0.878 | 0.831 | 0.757 | 0.837 |  |
| XGBoost (Internal validation cohort) | Sofa | 0.593 | 0.496, 0.690 | 0.383 | 0.400 | 0.657 | 0.367 |  |
|  | Model1 | 0.793 | 0.721, 0.866 | 0.535 | 0.511 | 0.763 | 0.561 |  |
|  | Model2 | 0.804 | 0.729, 0.879 | 0.614 | 0.600 | 0.799 | 0.628 |  |
|  | Model3 | 0.815 | 0.745，0.885 | 0.602 | 0.622 | 0.781 | 0.583 |  |
|  | Model4 | 0.778 | 0.702, 0.854 | 0.541 | 0.511 | 0.575 | 0.837 |  |
| LR (Test validation cohort) | Sofa | 0.705 | 0.616, 0.793 | 0.699 | 0.609 | 0.621 | 0.821 |  |
|  | Model1 | 0.761 | 0.680, 0.841 | 0.746 | 0.700 | 0.703 | 0.797 |  |
|  | Model2 | 0.778 | 0.700, 0.855 | 0.754 | 0.700 | 0.717 | 0.818 |  |
|  | Model3 | 0.813 | 0.740，0.886 | 0.788 | 0.744 | 0.752 | 0.838 |  |
|  | Model4 | 0.760 | 0.681, 0.838 | 0.743 | 0.689 | 0.703 | 0.805 |  |

| Supplementary Table S5. Modified Sequential organ failure assessment score (mSOFA). | | | | | | |
| --- | --- | --- | --- | --- | --- | --- |
| Variables | Scores | | | | | |
|  | 4 | 3 | | 2 | 1 | 0 |
| Lactate (mmol/L) | ≥6.1 | | 4.1-6.0 | 3.1-4.0 | 2.1-3.0 | ≤2.0 |
| Albumin (mg/dL) | ≤2.4 | | 2.5-3.0 | 3.1-3.5 | 3.6-4.0 | ≥4.1 |
| BUN (mg/dl) | ≥50.1 | | 40.1-50 | 30.1-40 | 20.1-30 | ≤20.0 |

**Supplementary Figures**


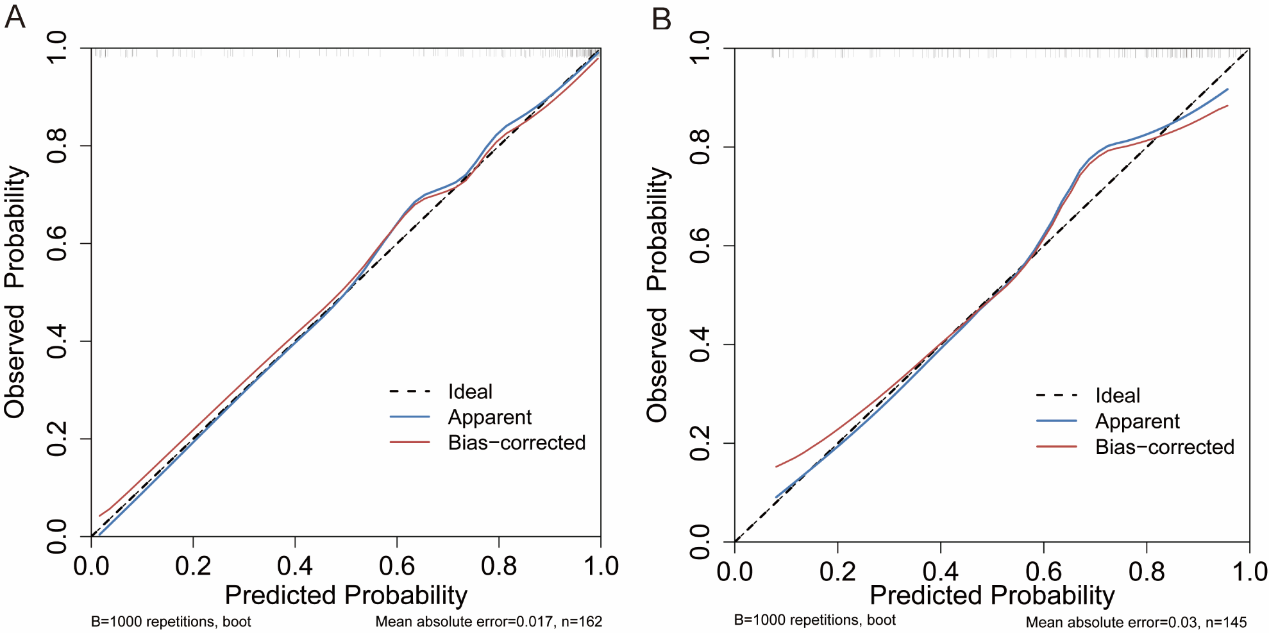
**Supplementary Figure S1.** Calibration curves of the mSOFA_3 model using Logistic Regression in internal validation (A) and test (B) cohorts. The dashed line represents the ideal calibration, where predicted probabilities match observed outcomes. In each graph, the blue line indicates apparent calibration, while the red line represents bias-corrected calibration after bootstrapping (B=1000 repetitions). For the internal validation cohort (A), the mean absolute error (MAE) is 0.017 with n=162. For the test cohort (B), the MAE is 0.03 with n=145.


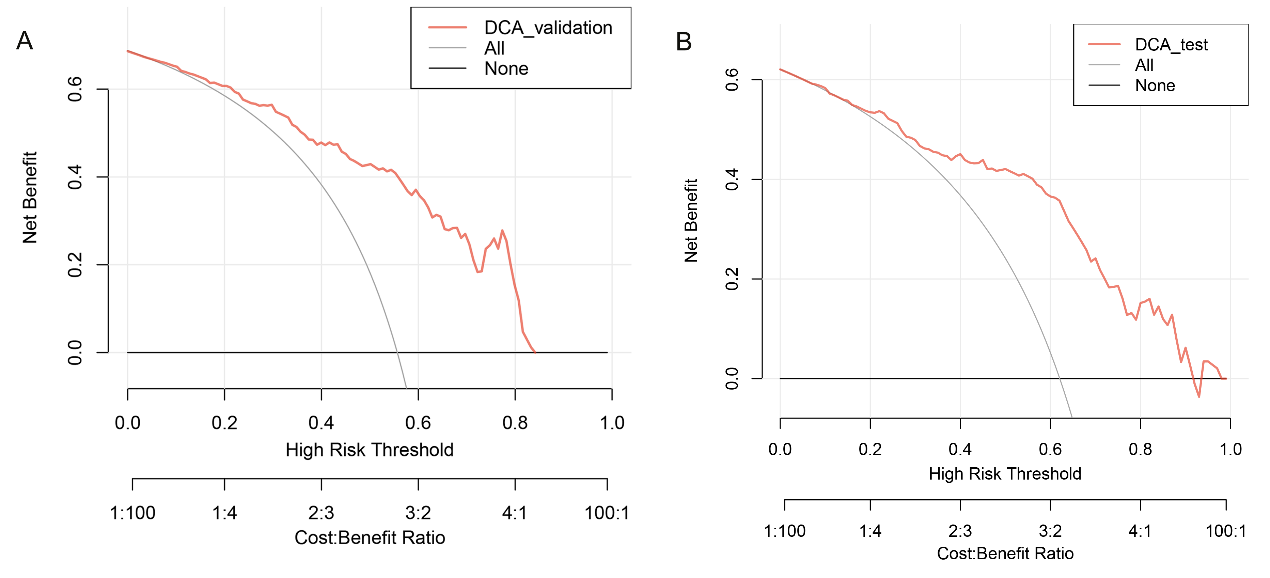


**Supplementary Figure S2.** Decision Curve Analysis (DCA) of the mSOFA_3 Model. (A) DCA for the internal validation cohort. (B) DCA for the test cohort.
